# Mucoid Degeneration of the Anterior Cruciate Ligament: A Case Report

**DOI:** 10.7759/cureus.50545

**Published:** 2023-12-14

**Authors:** Oussama El Alaoui, Ousama Jelti, Adnane Lachkar, Najib Abdeljaouad, Hicham Yacoubi

**Affiliations:** 1 Orthopaedics and Traumatology, University Hospital Mohammed VI, Oujda, MAR; 2 Orthopaedics, University Hospital Mohammed VI, Oujda, MAR

**Keywords:** mri- magnetic resonance imaging, degenerative knee, mucoid degeneration, anterior cruciate ligament (acl), arthroscopy

## Abstract

Mucoid degeneration (MD) is an uncommon pathological phenomenon that specifically affects the anterior cruciate ligament (ACL). This condition arises from the infiltration of yellowish material within the fibers of the ACL, contributing to the clinical presentation characterized by discomfort and limited mobility. MRI has proven to be the foremost diagnostic modality in effectively distinguishing MD of ACL from other potential pathologies. Preoperative recognition of this condition facilitates straightforward diagnosis, particularly via characteristic findings observed during knee arthroscopy. We present a case of MD of ACL, review prior studies about the condition, and outline its clinical features and symptoms, including those observed in our specific case.

## Introduction

Mucoid degeneration (MD) of the anterior cruciate ligament (ACL) is a rare condition, with a prevalence rate ranging from 0.2% to 1.2% [1]. The cause of this condition is not well understood and has been the subject of vigorous debate, with only a limited number of reports in the literature detailing its origin [2,3]. It is characterized by the infiltration of a mucoid-like substance within the ACL, which can cause knee pain and restricted motion [4]. Although it was once considered rare, recent reports suggest that it may be more common than previously thought, implying that it is underdiagnosed or misdiagnosed [5]. We describe a successful treatment approach for MD of ACL, which involves the application of arthroscopic debridement.

## Case presentation

**Figure 1:**
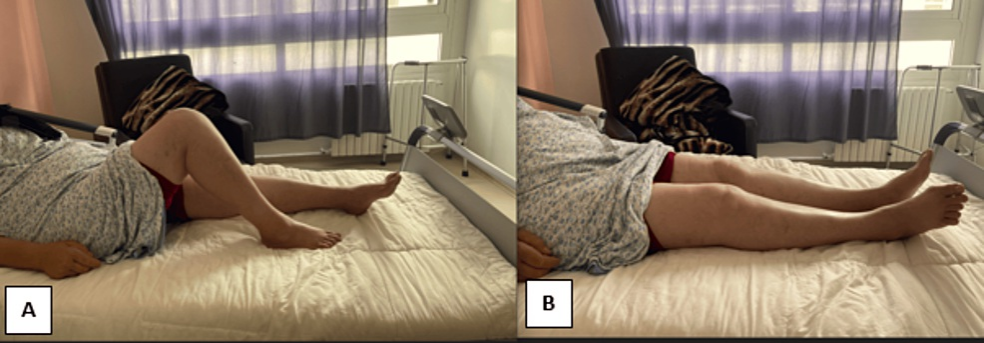
Examination of the patient at presentation A: Range of flexion of the right knee 90°. B: Range of extension of the right knee 0°

The patient was a 57-year-old female who presented to our hospital with a two-year history of right knee pain and limited flexion following a minor injury. A thorough examination was conducted. Notably, there was no apparent swelling or instability observed. The knee exhibited a restricted range of motion, spanning from 0° to 90°, with pain occurring during terminal flexion. Tenderness was localized to the lateral joint line of the knee, and the McMurray test yielded a positive result. Significantly, no clinical signs or symptoms suggestive of instability, ligamentous laxity, or patellofemoral pathology were evident (Figure 1).

**Figure 2:**
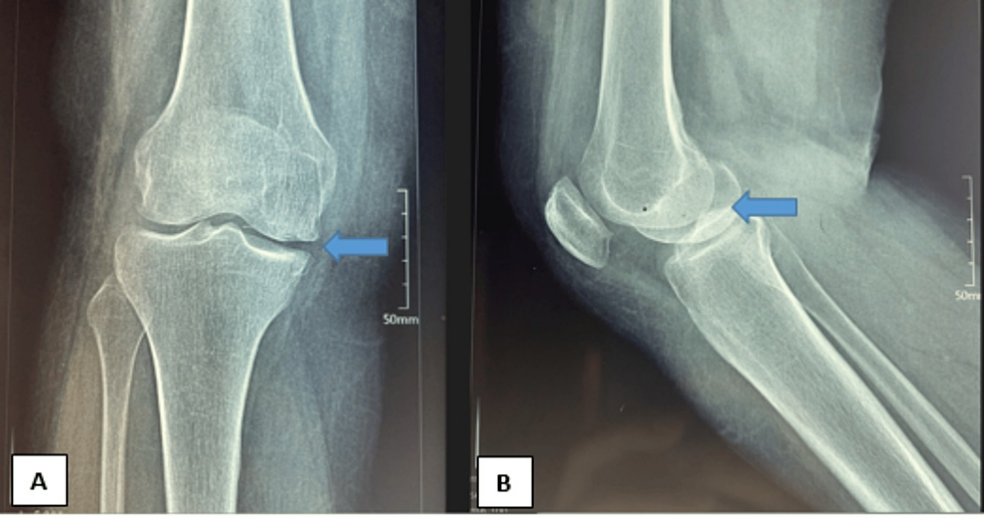
Plain radiographs show degenerative changes on the medial aspect of the knee A: Anteroposterior radiographs of the right knee. B: Lateral radiographs of the right knee

A plain X-ray examination provided valuable insights, revealing subtle degenerative changes, particularly in the medial compartment (Figure 2). The patient underwent conservative treatment for several months; however, there was no improvement in the condition.

**Figure 3:**
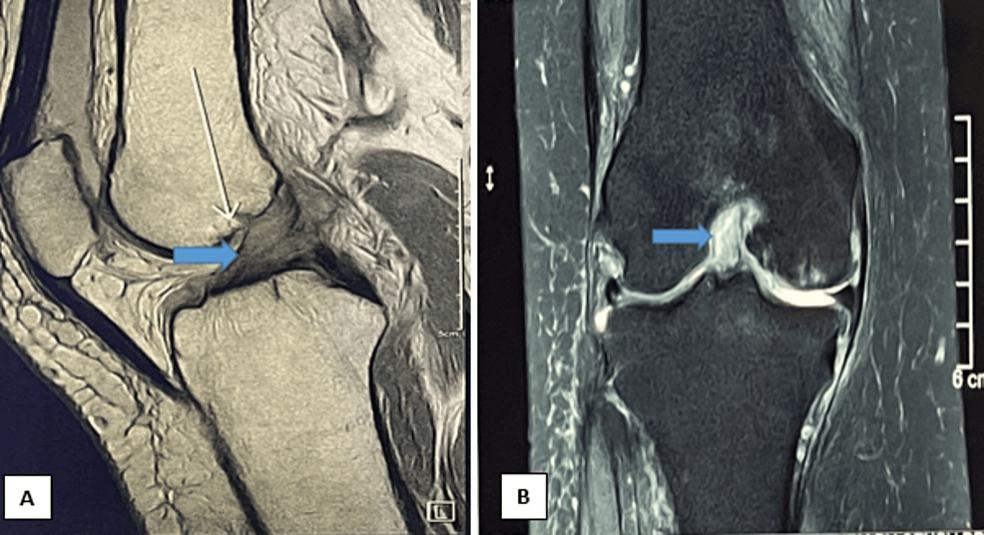
MRI of the knee at presentation A: MRI Sagittal T1-weighted image showing the high signal intensity of the anterior cruciate ligament with a "celery stalk" appearance. B: MRI Coronal T2-weighted image showing a thickening of the anterior cruciate ligament with meniscus lesions MRI: magnetic resonance imaging

MRI of the knee was performed, which revealed specific findings in the right knee. In the coronal view, a clear thickening of the ACL was evident, characterized by preserved fibers, and a consistent increase in signal intensity. Noteworthy findings on the sagittal view revealed an indistinct ACL presenting a distinctive "celery stalk" appearance (Figure 3).

**Figure 4:**
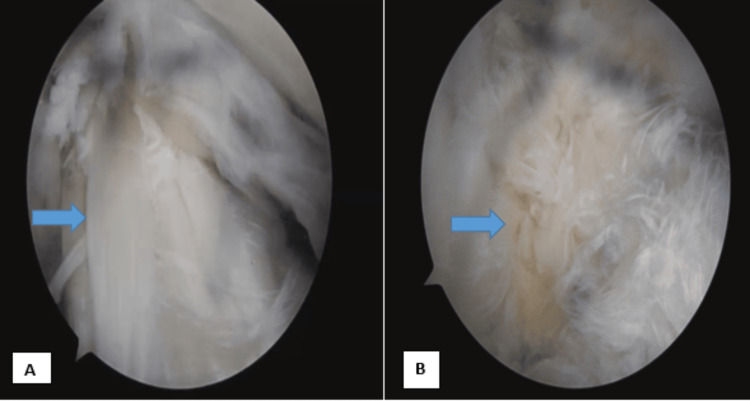
Arthroscopic images of the anterior cruciate ligament A: Enlarged anterior cruciate ligament occupying the intercondylar notch. B: Yellowish material and sclerotic lesions within the anterior cruciate ligament were subsequently removed through debridement

Arthroscopy was performed, uncovering femoropatellar arthritis marked by advanced osteocartilaginous lesions and degenerative changes in the lateral and medial meniscus. The continuous fibers, notably the anterolateral bundle, exhibited enlargement and displayed a yellowish degenerative appearance, confirming MD. Additionally, degenerative vertical lesions were evident in the external meniscus. A decision was made to perform debridement of the yellowish lesion of the ACL, and subsequent stability testing through the anterior drawer maneuver yielded satisfactory results (Figure 4). At the 10-month follow-up, the patient demonstrated a complete range of knee motion without experiencing pain or instability.

## Discussion

MD of ACL is an uncommon condition that leads to knee pain and discomfort, primarily impacting individuals in their fourth decade of life [6]. Kumar et al. [7] initially coined the term mucoid cystic degeneration to describe this condition affecting the ACL. It is characterized by significant disruption of the ligament's normal appearance and can contribute to knee pain. The predominant symptom associated with MD of ACL is knee pain, primarily located in the posterior aspect [8,9]. This pain is caused by mechanical impingement on the posterior cruciate ligament and the posterior capsule or bone erosions [9]. Additional clinical manifestations include a mechanical hindrance to extension, varying degrees of swelling, and audible clicking sounds [10].

MRI serves as a valuable tool for evaluating MD of ACL during preoperative assessment. It shows an ACL that is indistinctly defined, exhibiting an enlarged dimension while retaining a typical orientation, and presenting heightened signal intensity interspersed among visible intact ACL fibers, creating a distinctive "celery stalk" appearance [11]. On T1-weighted images, MD of ACL typically appears with intermediate signal intensity, while on T2-weighted images, it shows high signal intensity [11,12]. Hodler et al. correlated MRI appearances with histological findings and found that 29 out of 38 ligaments had focal areas of signal increase, suggesting a correlation between the focal MRI signal changes and the presence of degenerative changes in the ligaments [13].

Arthroscopic observations suggest that MD of ACL involves a hypertrophied, fibrillated ligament with the presence of yellowish mucinous material interspersed among the fibers. This is often accompanied by the absence of the ligamentum mucosum [12]. Additionally, a lack of smooth synovial lining is typically noted [14]. Arthroscopic surgery is a discretionary treatment option for MD of ACL. This procedure involves the partial removal of lesions within the ACL, leading to rapid pain relief and improved range of motion, without any persistent symptoms of instability. The reduction in volume and tension within the ACL is often attributed to the notable pain relief [15]. Debridement of mucinous substance, along with partial resection of ACL, is a recommended and effective therapy that does not cause instability, according to many authors [16].

While certain authors have underscored the significance of notchplasty as a supplementary step in the procedure, Motmans and Verheyden have challenged this idea by arguing against the necessity of notchplasty. They contend that a comprehensive debridement alone is sufficient to resolve impingement and effectively address the underlying pathology [4].

## Conclusions

The clinical and radiological characteristics of MD of ACL can be often inconclusive, potentially leading to a misdiagnosis of a torn ACL. Arthroscopists must be cognizant of this uncommon condition, as they may encounter a mucoid ACL during surgical procedures. This awareness is crucial for an accurate diagnosis and appropriate management of the condition.
